# Deciphering the Molecular Complexity of Hepatocellular Carcinoma: Unveiling Novel Biomarkers and Therapeutic Targets Through Advanced Bioinformatics Analysis

**DOI:** 10.1002/cnr2.2152

**Published:** 2024-08-08

**Authors:** Ata Moghimi, Nasrin Bani Hosseinian, Mahdi Mahdipour, Ehsan Ahmadpour, Alberto Miranda‐Bedate, Saeid Ghorbian

**Affiliations:** ^1^ Immunology Research Center, Tabriz University of Medical Sciences Tabriz Iran; ^2^ Stem Cell Research Center, Tabriz University of Medical Sciences Tabriz Iran; ^3^ Department of Applied Cell Sciences, Faculty of Advanced Medical Sciences Tabriz University of Medical Sciences Tabriz Iran; ^4^ Infectious and Tropical Diseases Research Center, Tabriz University of Medical Sciences Tabriz Iran; ^5^ Department of Data Science EURETOS Utrecht The Netherlands; ^6^ Department of Molecular Genetics Ahar Branch, Islamic Azad University Ahar Iran

**Keywords:** bioinformatics, hepatocellular carcinoma, hub genes, miRNAs

## Abstract

**Background:**

Hepatocellular carcinoma (HCC) represents a primary liver tumor characterized by a bleak prognosis and elevated mortality rates, yet its precise molecular mechanisms have not been fully elucidated. This study uses advanced bioinformatics techniques to discern differentially expressed genes (DEGs) implicated in the pathogenesis of HCC. The primary objective is to discover novel biomarkers and potential therapeutic targets that can contribute to the advancement of HCC research.

**Methods:**

The bioinformatics analysis in this study primarily utilized the Gene Expression Omnibus (GEO) database as data source. Initially, the Transcriptome analysis console (TAC) screened for DEGs. Subsequently, we constructed a protein–protein interaction (PPI) network of the proteins associated to the identified DEGs with the STRING database. We obtained our hub genes using Cytoscape and confirmed the results through the GEPIA database. Furthermore, we assessed the prognostic significance of the identified hub genes using the GEPIA database. To explore the regulatory interactions, a miRNA‐gene interaction network was also constructed, incorporating information from the miRDB database. For predicting the impact of gene overexpression on drug effects, we utilized CANCER DP.

**Results:**

A comprehensive analysis of HCC gene expression profiles revealed a total of 4716 DEGs, consisting of 2430 upregulated genes and 2313 downregulated genes in HCC sample compared to healthy control group. These DEGs exhibited significant enrichment in key pathways such as the PI3K‐Akt signaling pathway, nuclear receptors meta‐pathway, and various metabolism‐related pathways. Further exploration of the PPI network unveiled the P53 signaling pathway and pyrimidine metabolism as the most prominent pathways. We identified 10 hub genes (*ASPM*, *RRM2*, *CCNB1*, *KIF14*, *MKI67*, *SHCBP1*, *CENPF*, *ANLN*, *HMMR*, and *EZH2*) that exhibited significant upregulation in HCC samples compared to healthy control group. Survival analysis indicated that elevated expression levels of these genes were strongly associated with changes in overall survival in HCC patients. Lastly, we identified specific miRNAs that were found to influence the expression of these genes, providing valuable insights into potential regulatory mechanisms underlying HCC progression.

**Conclusion:**

The findings of this study have successfully identified pivotal genes and pathways implicated in the pathogenesis of HCC. These novel discoveries have the potential to significantly enhance our understanding of HCC at the molecular level, opening new ways for the development of targeted therapies and improved prognosis evaluation.

**Abbreviations**
CCNB1: Cyclin B1
DEGs: differentially expressed genes
GEO: Gene Expression Omnibus
GEPIA: Gene Expression Profiling Interactive Analysis
GTEx: Genotype‐Tissue Expression
HCC: hepatocellular carcinoma
OS: overall survival
PPI: protein–protein interaction
RRM2: ribonucleotide reductase regulatory subunit M2
TAC: transcriptome analysis console
TCGA: Cancer Genome Atlas

## 1 Introduction

Liver cancer is a significant global health burden. In the year 2020, approximately 905 700 people were diagnosed with liver cancer, and during the same year, 830 200 individuals lost their lives due to it. Liver cancer is among the top five causes of death worldwide, according to the data from 90 countries. It is estimated that this figure will increase by more than 55.0% between 2020 and 2040, meaning the mortality rate will reach 1.3 million people, and the incidence rate will rise to 1.4 million people [1]. Hepatocellular carcinoma (HCC) is the most common type of primary malignant liver tumor, accounting for approximately 85%–90% of cases [2]. Localized HCC has a poor prognosis, with a 5‐year overall survival (OS) rate of only 30%, which drops to less than 5% when distant metastases are present [3]. Liver resection is often effective for early‐stage HCC, but less than 30% of patients are eligible for surgery, and a majority of them experience tumor recurrence within a few years [4]. Despite advancements in cancer biology and genetic profiling, there is still much to learn about the molecular causes of HCC. A comprehensive understanding of HCC pathogenesis is crucial for improving early detection and treatment strategies, and thereby enhancing patient survival rates. To achieve this, the utilization of diverse bioinformatic tools is necessary to identify key genes and biological pathways that drive tumor growth and progression [5, 6].

Identifying suitable biomarkers for cancer, such as HCC, holds immense potential for advancing research and clinical practice in this field. The work of Nault et al. in discovering biomarkers for HCC exemplifies how such findings can shift researchers' perspectives and significantly impact the field. Bioinformatics emerges as a powerful tool in this endeavor, enabling the identification of effective biomarkers, elucidation of cellular pathways, and prediction of their impact on cancer progression [7]. Several established biomarkers such as Telomerase reverse transcriptase (TERT), tumor protein p53 (TP53), Catenin Beta 1 (CTNNB1), AT‐rich interaction domain 1A (ARID1A), and Axis inhibition protein 1 (AXIN1) have been validated for their impact on HCC and related pathways, including PI3K/AKT/mTOR and RAS/RAF/MAPK pathways [8]. However, our understanding of HCC pathogenesis remains limited due to constraints such as small sample sizes. It is highly likely that additional biomarkers and pathways play a significant role in HCC progression. An integrated bioinformatics study utilizing the latest genomic data represents a pivotal advancement in cancer research. This approach addresses the limitations posed by small sample sizes and inconsistent methodologies, offering a comprehensive exploration of HCC biology. By integrating diverse omics data and employing rigorous analytical methods, this study has the potential to unveil novel biomarkers and pathways associated with HCC. Moreover, overcoming these limitations is essential for revolutionizing our understanding of cancer biology and facilitating the development of more effective treatments. By elucidating the complex molecular landscape of HCC, integrated bioinformatics studies pave the way for personalized therapeutic interventions tailored to individual patients, ultimately improving clinical outcomes in the fight against HCC.

In our research, we used the microarray dataset GSE45267 from the Gene Expression Omnibus (GEO) to examine the variations in gene expression between HCC and adjacent noncancerous tissues. Computational tools such as STRING and Cytoscape were utilized to construct PPI networks and pinpoint hub genes associated with HCC. Furthermore, we conducted survival analyses and explored drug‐gene interactions to identify critical genes and pathways influencing the pathogenesis and prognosis of HCC. These discoveries hold great potential for advancing the field of diagnostic and therapeutic strategies for HCC.

## 2 Material and Methods

### 2.1 Data Source and Identification of DEGs

In this study, we utilized microarray data obtained from the GEO repository (accessible at https://www.ncbi.nlm.nih.gov/gds). Our focus was on the gene expression profiles of human HCC based on the following criteria: (a) Analysis type: Cancer versus regular; (b) Cancer type: HCC; (c) Data type: mRNA; (d) Sample type: Clinical specimen; (e) Microarray platform: Affymetrix Human Genome U133 Plus 2.0 Array (GPL570). For the present work, GSE45267 dataset was selected, which consisted on 87 gene expression profiles derived from tissue samples, including 48 primary HCC samples and 39 noncancerous tissues, from a cohort of 61 patients. It is important to note that the HCC tissues were collected from 16 young HCC patients and 32 older HCC patients. To ensure data reliability and relevance, we chose 7 human HCC samples from young patients and 13 human normal liver samples from young HCC patients. To identify the differentially expressed genes (DEGs) between HCC and normal liver tissues, the Transcriptome analysis console (TAC) from GEO was employed with a threshold of logarithmic Fold Change (log FC) >2 and an adjusted *p* value <0.01 to define the DEGs.

### 2.2 PPI Network and Modular Analysis

To assess the significance of the DEGs identified in this study, we constructed a PPI network with their associated proteins. This network analysis allowed us to examine the interactions between different DEGs and determine their functional importance. For this purpose, we utilized the STRING database (accessible at https://string‐db.org/) Version: 12.0.

### 2.3 Identification of Candidate Genes

To construct and visualize the PPI networks, we employed Cytoscape software, version 3.6.0 [9]. To identify significant modules of hub genes within the PPI network, we employed the Molecular Complex Detection (MCODE) plug‐in in Cytoscape that enables the identification of clusters of highly interconnected (hub) genes within the PPI network. In our study, the selection of significant hub genes within the PPI network was based on a degree cutoff of a minimum of 99 connections with other nodes in the network and betweenness centrality >0.022. Nodes with higher betweenness centrality values were considered more critical in maintaining efficient information flow within the network. Additionally, we employed a closeness centrality threshold >0.104, where nodes with higher values were deemed more central in terms of their proximity to other nodes in the network.

### 2.4 Analysis of Signaling Pathways

The *Enrichr* database (https://maayanlab.cloud/Enrichr/) was used to identify the cellular pathways in which our target genes are involved [10, 11]. We used KEEG pathway resource to obtain and represent our results. A threshold of the top 10 pathways was applied as a cutoff point in the analysis.

### 2.5 GEPIA Analysis of Gene Expression

The evaluation of gene expression was conducted through the utilization of the Gene Expression Profiling Interactive Analysis (GEPIA) tool. GEPIA performs a comparative analysis of gene expression across diverse cancer types and normal tissues based on data from The Cancer Genome Atlas (TCGA) and the Genotype‐Tissue Expression (GTEx) projects [12]. In the box plot analysis, a cutoff of |Log2FC| ≥ 1 and a *p*‐value cutoff of ≤0.01 were employed, with a jitter size set to 0.4 to enhance data visualization. For survival plots, group cutoffs were determined based on the median, with high and low cutoff percentages set at 50%. Hazard ratios were calculated using the Cox proportional hazards (PH) model, with 95% confidence intervals added as dotted lines. Time units on the axis were standardized to months.

### 2.6 Analysis of Gene‐Disease Association

A comprehensive analysis of gene‐disease associations was performed through the utilization of DisGeNET (accessible at https://www.disgenet.org/). This platform allows us to explore the intricate connections between specific genes and a wide array of diseases in various pathological conditions. We focused on identifying 10 top diseases that were associated with the upregulated genes identified in our study.

### 2.7 Prediction of Pharmacological Targets

By feeding the expression levels of the hub genes into *cancerDP* tool (https://webs.iiitd.edu.in/raghava/cancerdp/about.php), we can identify potential drugs targeting these genes in a concrete cancer type, and classified those drugs as potentially ineffective (resistant) or effective (sensitive) against the specific combination of cancer type—hub gene expression levels. miRDB database (http://www.mirdb.org/) was used to identify miRNAs that target these hub genes.

## 3 Results

### 3.1 The Identification of DEGs in BC

**FIGURE 1:**
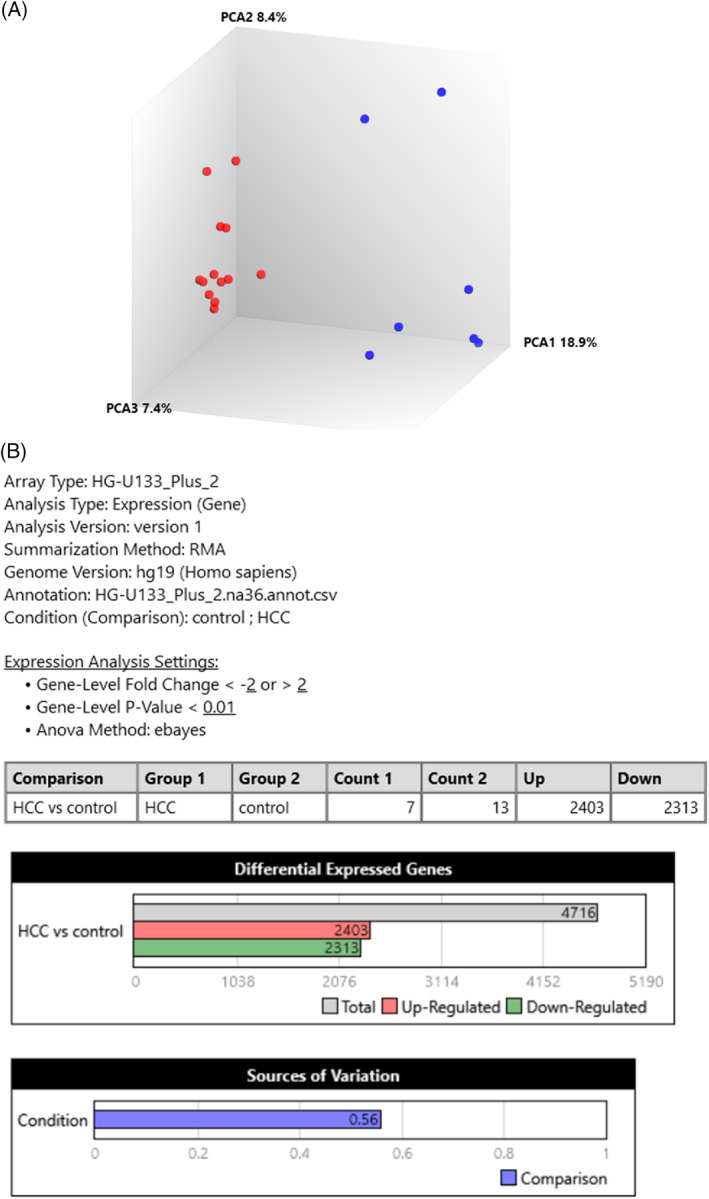
Gene expression analysis of GSE45267 dataset. (A) Principal component analysis (PCA) displays normal human liver samples as red circles and human HCC samples as blue circles, showing a clear clusterization of the two groups. (B) Differential gene expression analysis of HCC versus healthy samples shows 2403 genes upregulated and 2313 genes downregulated, being tumoral condition the major source of variability. HCC, hepatocellular carcinoma.

PCA analysis of the GSE45267 dataset showed a different expression profile between tumor and healthy samples (Figure 1A). Table 1 provides a total of 100 top fold change DEGs up‐ and down‐ regulated explaining these differences. With the TAC software, we concluded that out of 54 613 genes, 4716 genes have changed their expression level, from which 2403 genes were upregulated and 2313 genes were downregulated (Figure 1B). The tumoral condition moreover was the major source of variability (Figure 1B).

**TABLE 1:** Top 100 fold‐change genes that are up‐ and down‐regulated in HCC.

Type	Gene
Upregulated genes	GPC3, SPINK1, LINC01296, CD24, PEG10, ECAM, TOP2A, CTHRC1, DCDC2, HKDC1, RRM2, SULT1C2, ASPM, HIST1H2BG, NEB, GINS1, CRNDE, SOX4, IGF2BP3, DKK1, ANLN, CDK1, HMMR, BEX2, DTL, PPP1R9A, KRT19, PRR11, CENPF, B3GNT5, NEK2, KIAA0101, VCAN, ECT2, ITGA2, RAGAP1, PRC1, ZWINT, AURKA, HS6ST2, CCNB1, SPINT1, CTNND2, CDKN3, CDC6, KIF4A, C12orf75, CDC20, ACSL4, BUB1B, NUF2, UHRF1, CLGN, COCH, HK2, GMNN, SFN, HELLS, FABP5, FOXQ1, CCL20, NDC80, CDC7, BIRC5, FAM72A; FAM72B; FAM72C; FAM72D, PRAME, FRAS1, TTK, NUSAP1, PBK, GTSE1, SPP1, CENPU, COL15A1, FAM83D, PLPP2, PKM, KIF11, CENPA, MCM6, AKR1B10, C1orf186; LOC100505650, GAS2L3, ROBO1, PLEKHB1, ASNS, SLC6A8, LOC728715; OVOS; OVOS2, C1orf106, HOXA10, NCAPG, TPX2, MAP7D2, TYMS, COL1A2, CENPW, FOXM1, FEN1, DLGAP5
Downregulated genes	ADH4, CYP2E1, HPD, C9, HAMP, CPS1, PCK1, MT1M, GYS2, SLC22A1, SULT2A1, CYP1A2, APOF, SLCO1B3, LINC00844, SLC10A1, CYP8B1, THRSP, CYP2A6, LECT2, ADH1C, HPR, ADH1B, CFHR4, CYP3A4, ADRA1A, CYP4A11; CYP4A22, F9, GLYAT, HAO2, FCN3, CYP2C8, CXCL14, IGH; IGHA1; IGHA2, SAA1; SAA2; SAA2, SAA4, GLYATL1; LOC100287413, LINC01093, OIT3, HGFAC, AKR1D1, GBA3, CYP2C9, FCN2, DNASE1L3, FETUB, ETNPPL, GNMT, DHRS2, NAT2, CFHR3, HSD17B6, NNMT, SLC22A7, PLG, ALDOB, C8A, CRHBP, CYP39A1, SLC27A5, GLS2, CLEC1B, SLC51A, LPA, MT1F, C3P1, TTC36, RDH16, CYAT1; IGLC1; IGLC2; IGLC3; IGLJ3; IGLV144; IGLV325; IGLV43, ANG, CLEC4G, A1BGCYP1A2, OTC, CNDP1, ACSM2A; ACSM2B, 13, ALDH1L1, SAA4IGHG1; IGHG3; IGHM; IGHV431, IGF1, ESR1, MASP2, SLC1A2, HRG, AQP9, KCNN2, SLC25A47, MOGAT2, SERPINC1, SLC25A18, NR1I2, SPP2, ADH1A, HSD11B1, C6, ABCA8, CYP2C19, RTP3, KMO, MBL2, JCHAIN, IGK; IGKC; IGKV1‐5; IGKV2‐24, C7

Abbreviation: HCC, hepatocellular carcinoma.

### 3.2 Identification of Key Candidate DEGs by PPI Network Analysis

**FIGURE 2:**
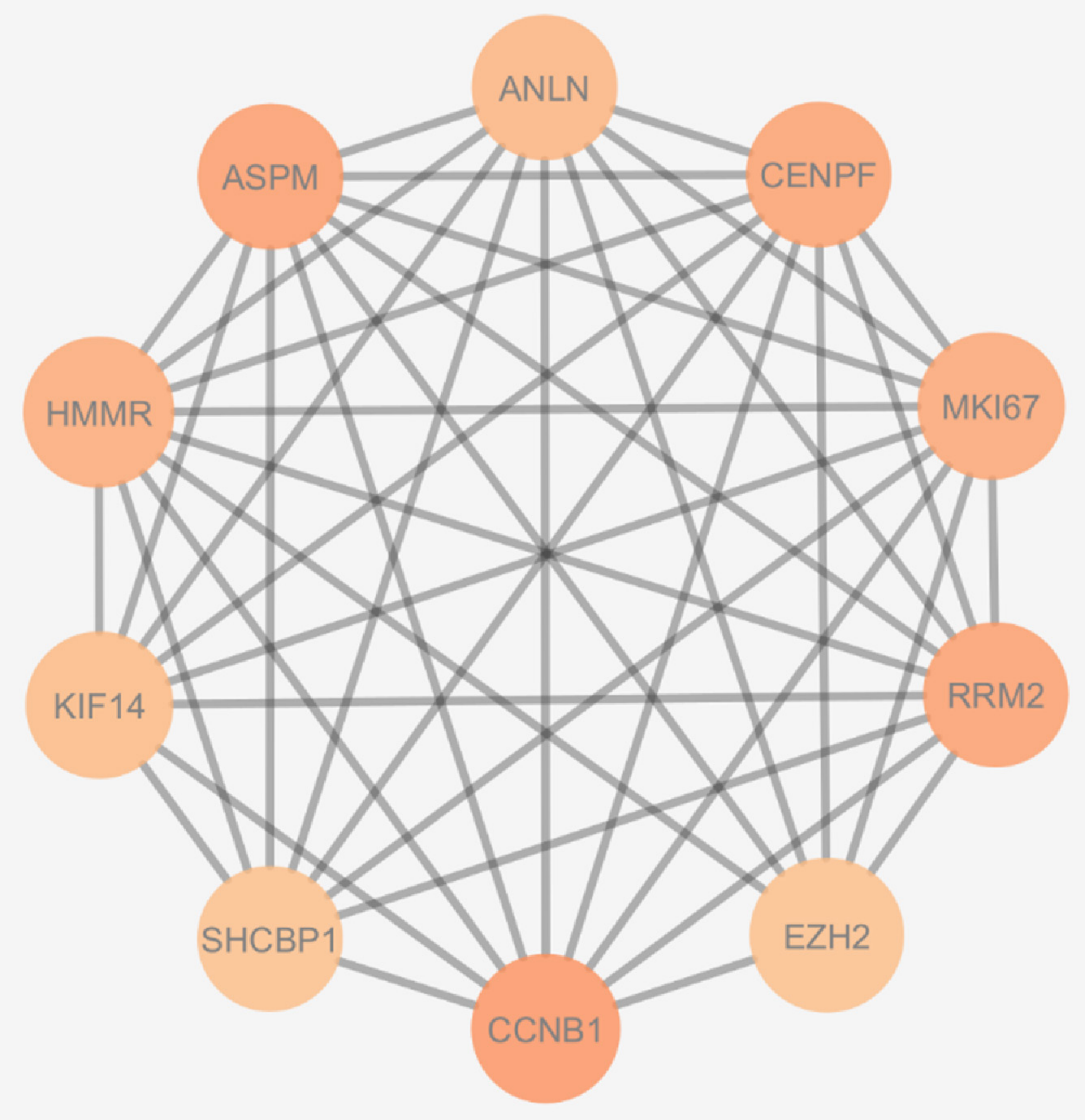
Connectivity between the top 10 hub genes (ASPM, RRM2, CCNB1, KIF14, MKI67, SHCBP1, CENPF, ANLN, HMMR, EZH2). The degree increases from light red to darker red. (Degree ≥99, betweenness centrality ≥0.022, closeness centrality ≥0.104.)

Using Cytoscape, we selected from the up‐regulated genes those having a degree ≥99, betweenness centrality ≥0.022 and closeness centrality ≥0.104, to pinpoint the highly interconnected DEGs (hub genes). Only *ASPM, RRM2, CCNB1, KIF14, MKI67, SHCBP1, CENPF, ANLN, HMMR, EZH2* remained (Figure 2).

### 3.3 Analysis of Signaling Pathways

**FIGURE 3:**
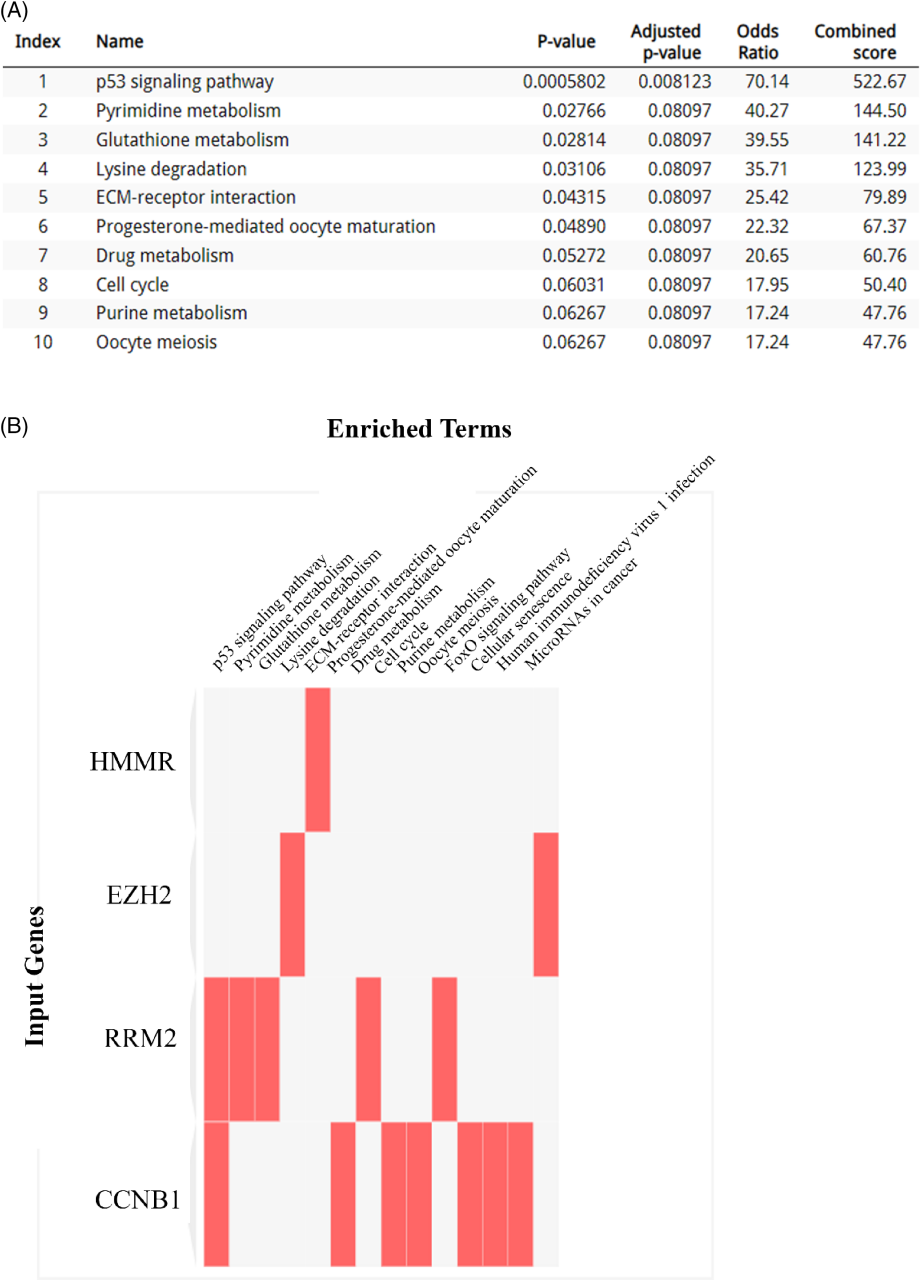
KEGG pathway enrichment analysis of DEGs in HCC (Adj *p*‐value <0.05). (A) Top10 cellular pathways that are associated with the top10 hub genes. (B) Impact of hub genes on the mentioned cellular pathways. Four hub genes (RRM2, HMMR, CCNB1, and EZH2) are predicted to be significantly impacting one or more pathways (in red in the heatmap). DEGs, differentially expressed genes; HCC, hepatocellular carcinoma.

*Enrichr* tool with the KEGG data base showed that the p53 signaling pathway, pyrimidine metabolism, glutathione metabolism, lysine degradation, ECM‐receptor interaction and cell cycle are the tumor related pathways where these hub genes have an important role (Figure 3A). Importantly, the hub genes that have the most significant impact on these pathways are HMMR, EZH2, RRM2, and CCNB1 (Figure 3B).

### 3.4 Analysis of Core Genes by the Kaplan Meier Plotter and GEPIA

**FIGURE 4:**
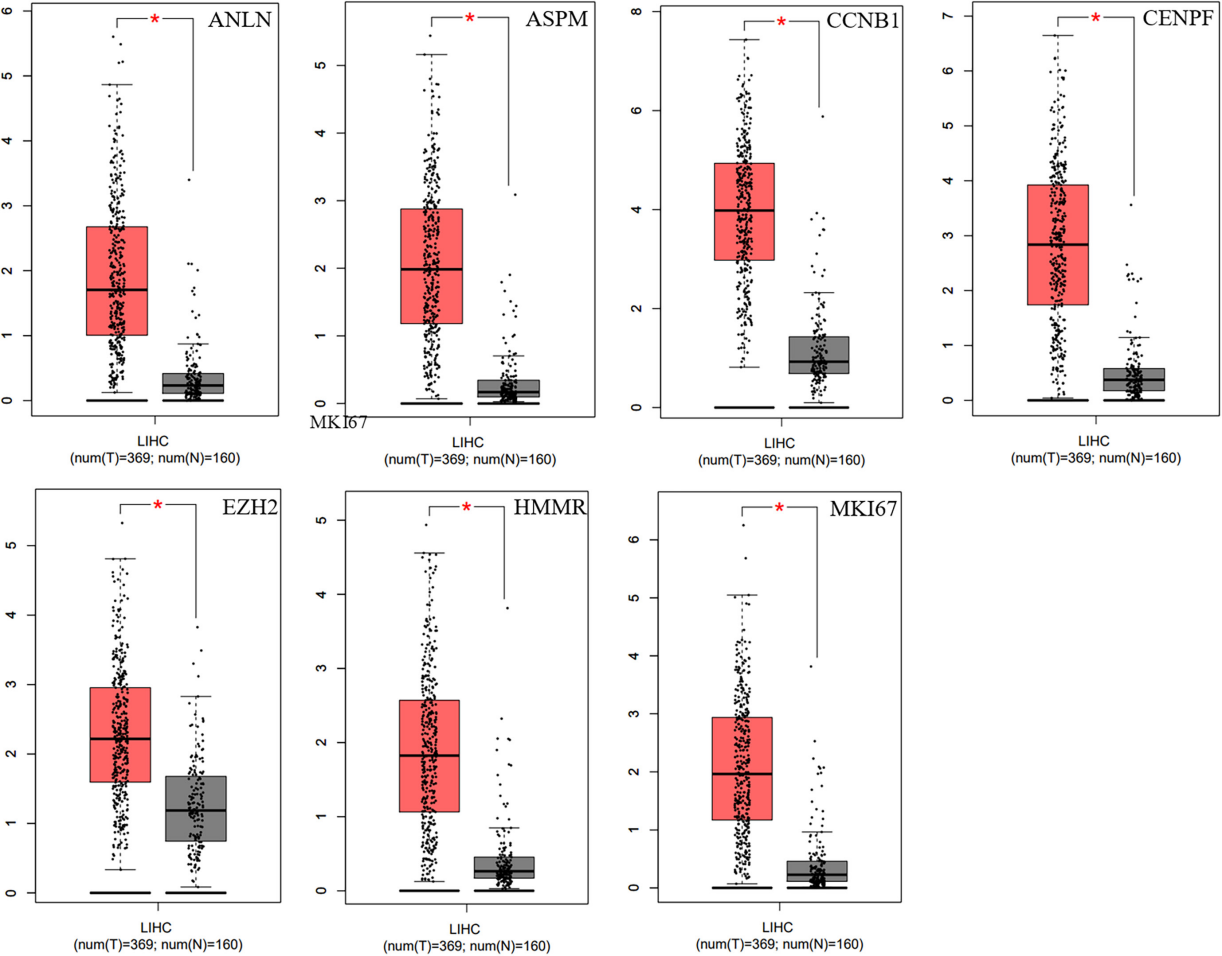
Gene expression of ASPM, CCNB1, CENPF, EZH2, MKI67, ANLN, HMMR, RRM2, KIF14, and SHCBP1 in liver hepatocellular carcinoma (LIHC) using the GEPIA tumor database. In all cases except KIF14, and SHCBP1 LIHC samples exhibited significantly higher gene expression compared to normal tissue samples; T, tumor tissues (red); N, normal tissues (gray). *p < 0.01.

**FIGURE 5:**
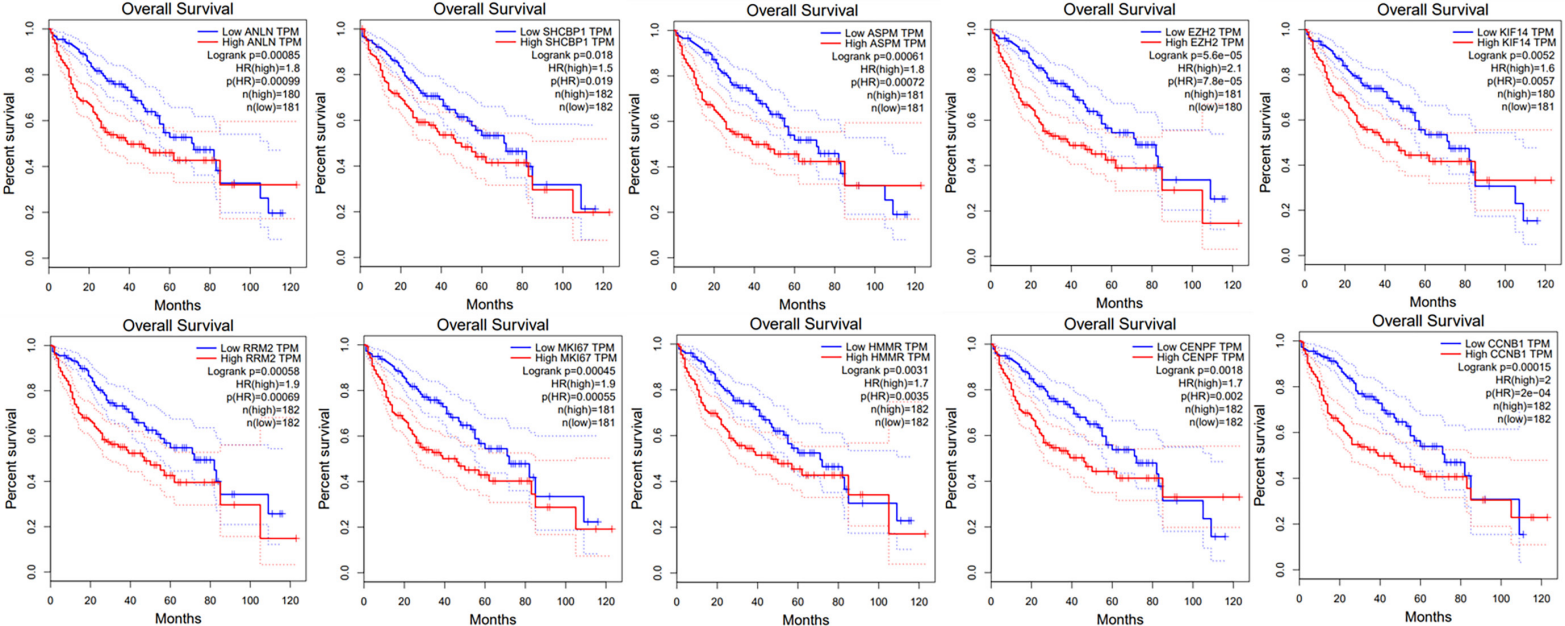
Survival analysis for the top 10 hub genes. High expression of RRM2, SHCBP1, HMMR, MKI67, and EZH2 was linked with the reduction of overall survival (OS) (*p* = 0.00058, *p* = 0.018, *p* = 0.0031, *p* = 0.00045, and *p* = 5.6e−05 respectively), while high expression of ANLN, ASPM, CCNB1, CENPF, and KIF67 were associated with improved OS (*p* = 0.00085, *p* = 0.00061, *p* = 0.00015, *p* = 0.0018, and *p* = 0.0052, respectively).

In line with our predictions, GEPIA tumor database revealed that *ASPM*, *CCNB1*, *CENPF*, *EZH2*, *MKI67*, *ANLN*, *HMMR*, and *RRM2* exhibited significantly elevated levels in HCC compared to their expression in healthy tissues (*p*‐value Cutoff: 0.01) (Figure 4), suggesting a key role of these genes in HCC. The GEPIA survival analysis from the top10 hub gene list revealed that high expression of *RRM2*, *SHCBP1*, *HMMR*, *MKI67*, and *EZH2* was linked with a reduction of the OS to HCC (*p* = 0.00058, *p* = 0.018, *p* = 0.0031, *p* = 0.00045, and *p* = 5.6e−05 respectively), while high expression of ANLN, ASPM, CCNB1, CENPF, and MKI67 was associated with improved OS (*p* = 0.00085, *p* = 0.00061, *p* = 0.00015, *p* = 0.0018, and *p* = 0.0052, respectively) (Figure 5).

### 3.5 Gene‐Diseases Association

Our exploration of DisGeNET uncovered a lot of information regarding the genetic factors that underlie, between others, several cancer types. The gene‐disease association details, along with their potential implications, are meticulously documented in Table 2. Importantly, the four most important hub genes *RRM2*, *HMMR*, *EZH2*, and *CCNB1* appeared to play a significant role in various types of cancer such as non‐small cell lung carcinoma (NSCLC), liver and breast cancer. Additionally, *HMMR* is related to prostate cancer, while *RRM2* and *CCNB1* shared association with colorectal cancer.

**TABLE 2:** This table provides the connection between the top 10 hub genes and diseases (*CancerDP*) and the miRNAs potentially targeting them (*miRDB*).

Gene	Description	Diseases	miRNA	Drugs	
				Drugs with resistance	Drugs with sensitivity
aspm	Abnormal spindle microtubule assembly	Autosomal Recessive Primary Microcephaly Microcephaly, Primary Autosomal Recessive, 5 Liver carcinoma Adenoid Cystic Carcinoma Malignant neoplasm of salivary gland Polycystic Ovary Syndrome Sclerocystic Ovaries Congenital microcephaly Severe intellectual disability Global developmental delay	hsa‐miR‐10 522‐5p hsa‐miR‐4307 hsa‐miR‐3646 hsa‐miR‐3662 hsa‐miR‐3613‐5p hsa‐miR‐382‐5p hsa‐miR‐425‐5p hsa‐miR‐1252‐3p hsa‐miR‐548c‐3p	Lapatinib Erlotinib AEW541 Sorafenib PLX4720 Panobinostat Paclitaxel AZD0530 PD0332991 Topotecan Irinotecan L685458 Nilotinib RAF265 TKI258 17AAG PD0325901 PHA665752	ZD6474 Nutlin3 LBW242 AZD6244 TAE684 PF2341066
EZH2	Enhancer of zeste 2 polycomb repressive complex 2 subunit	Weaver syndrome Diffuse Large B‐Cell Lymphoma Osteosarcoma Prostatic Neoplasms Myelodisplastic Syndrome Liver carcinoma Breast Carcinoma Endometrial Carcinoma Primary Myelofibrosis Leukemia, Myelomonocytic, Chronic	hsa‐miR‐101‐3p hsa‐miR‐548aw hsa‐miR‐217‐5p hsa‐miR‐6807‐3p hsa‐miR‐605‐3p hsa‐miR‐527 hsa‐miR‐518a‐5p hsa‐miR‐653‐3p hsa‐miR‐144‐3p hsa‐miR‐26b‐5p	N/A	N/A
RRM2	Ribonucleotide reductase M2	Colorectal Carcinoma Liver carcinoma Adenocarcinoma of lung (disorder) Polycystic Ovary Syndrome Sclerocystic Ovaries Bladder Neoplasm Non‐Small Cell Lung Carcinoma Breast Carcinoma Glioblastoma Pancreatic carcinoma	hsa‐miR‐4666a‐3p hsa‐miR‐664a‐3p hsa‐miR‐342‐3p hsa‐miR‐6816‐3p hsa‐miR‐4328 hsa‐miR‐6504‐3p hsa‐miR‐10 399‐5p hsa‐miR‐1284 hsa‐miR‐106b‐5p hsa‐miR‐10 398‐5p	Panobinostat Lapatinib AEW541 TKI258 PLX4720 Sorafenib AZD0530 Topotecan PHA665752 Erlotinib RAF265 Irinotecan	Paclitaxel ZD6474 17AAG AZD6244 LBW242 Nutlin3 Nilotinib PD0332991 PD0325901 L685458 PF2341066 TAE684
CCNB1	Cyclin B1	Liver carcinoma Polycystic Ovary Syndrome Squamous cell carcinoma of the head and neck Diabetes Mellitus, Experimental Visual seizure Breast Carcinoma Colorectal Carcinoma Glioblastoma Stomach Carcinoma Non‐Small Cell Lung Carcinoma	hsa‐miR‐548n hsa‐miR‐559 hsa‐miR‐548ar‐5p hsa‐miR‐548as‐5p hsa‐miR‐548w hsa‐miR‐548i hsa‐miR‐548ab hsa‐miR‐548a‐5p hsa‐miR‐548ay‐5p hsa‐miR‐548 h‐5p	TKI258 AZD0530 L685458 Sorafenib AEW541 PD0332991 Panobinostat PHA665752 Paclitaxel Lapatinib RAF265 Topotecan Irinotecan Nilotinib Nutlin3 PD0325901 PLX4720	17AAG PF2341066 ZD6474 AZD6244 TAE684 Erlotinib LBW242
KIF14	Kinesin family member 14	MECKEL SYNDROME 12 Microcephaly Liver carcinoma Intrauterine growth restriction (IUGR) Meckel syndrome type 1 Global developmental delay Blindness Ureteral hypoplasia Cerebral ventriculomegaly Attention deficit hyperactivity disorder	hsa‐miR‐3646 hsa‐miR‐1976 hsa‐miR‐607 hsa‐miR‐6515‐3p hsa‐miR‐126‐5p hsa‐miR‐340‐5p hsa‐miR‐4256 hsa‐miR‐5692a hsa‐miR‐11 181–5p hsa‐miR‐4477a	Sorafenib Topotecan PLX4720 Panobinostat Lapatinib Paclitaxel Erlotinib AZD0530 PHA665752 AEW541 PD0332991 ZD6474 RAF265	Irinotecan 17AAG AZD6244 PD0325901 Nutlin3 TKI258 L685458 Nilotinib PF2341066 TAE684 LBW242
MKI67	Marker of proliferation Ki‐67	Liver carcinoma Breast Carcinoma Adenocarcinoma Colorectal Carcinoma Ovarian neoplasm Psoriasis Mesenchymal Chondrosarcoma Glioblastoma Stomach Carcinoma Non‐Small Cell Lung Carcinoma	hsa‐miR‐7154‐3p hsa‐miR‐6806‐3p hsa‐miR‐3135b hsa‐miR‐3928‐5p hsa‐miR‐129‐2‐3p hsa‐miR‐129‐1‐3p hsa‐miR‐6500‐3p hsa‐miR‐3614‐5p hsa‐miR‐187‐5p hsa‐miR‐4673	Erlotinib Sorafenib Nilotinib Panobinostat RAF265 Paclitaxel Irinotecan Topotecan PLX4720 ZD6474 Lapatinib	TKI258 17AAG AEW541 PD0325901 AZD6244 Nutlin3 AZD0530 PHA665752 PF2341066 TAE684 PD0332991 L685458 LBW242
HMMR	Hyaluronan‐mediated motility receptor (RHAMM)	Breast Carcinoma Myeloid Leukemia, Chronic Liver carcinoma Polycystic Ovary Syndrome Multiple Myeloma Prostate carcinoma Glioblastoma Multiforme Adenocarcinoma of lung (disorder) Non‐Small Cell Lung Carcinoma Endometrial Carcinoma	hsa‐miR‐548au‐3p hsa‐miR‐7854‐3p hsa‐miR‐122b‐5p hsa‐miR‐7853‐5p hsa‐miR‐105‐5p hsa‐miR‐3920 hsa‐miR‐892a hsa‐miR‐4330 hsa‐miR‐7155‐5p hsa‐miR‐8077	AEW541 Paclitaxel ZD6474 AZD0530 PLX4720 Panobinostat Topotecan TKI258 Irinotecan Lapatinib 17AAG Nutlin3 RAF265 PD0332991 PHA665752 PD0325901 Nilotinib	Erlotinib Sorafenib LBW242 PF2341066 L685458 TAE684 AZD6244
SHCBP1	SHC SH2‐domain binding protein 1	Synovial sarcoma Liver Cirrhosis, Experimental Secondary malignant neoplasm of bone Phenylketonurias Liver carcinoma Adult Synovial Sarcoma Childhood Synovial Sarcoma Carcinoma of lung Glioma Malignant neoplasm of colon and/or rectum	hsa‐miR‐3163 hsa‐miR‐4482‐3p hsa‐miR‐545‐3p hsa‐miR‐197‐3p hsa‐miR‐34a‐3p hsa‐miR‐19a‐3p hsa‐miR‐9902 hsa‐miR‐19b‐3p hsa‐miR‐889‐3p hsa‐miR‐5011–5p	Lapatinib PHA665752 Topotecan Sorafenib AEW541 Erlotinib AZD0530 LBW242 PLX4720 Irinotecan Paclitaxel	RAF265 17AAG Panobinostat TKI258 PD0332991 PD0325901 Nutlin3 ZD6474 Nilotinib PF2341066 TAE684 L685458 AZD6244
CENPF	Centromere protein F	Anomalies Microcephaly Malignant neoplasm of prostate Liver carcinoma Breast Carcinoma Prostatic Neoplasms Polydactyly Chronic myeloproliferative disorder Mammary Carcinoma, Human Seckel syndrome	hsa‐miR‐338‐5p hsa‐miR‐4521 hsa‐miR‐4744 hsa‐miR‐19a‐5p hsa‐miR‐4279 hsa‐miR‐19b‐2‐5p hsa‐miR‐19b‐1‐5p hsa‐miR‐6835‐3p hsa‐miR‐874‐3p hsa‐miR‐3689f	AZD0530 Lapatinib Paclitaxel Sorafenib PLX4720 L685458 TKI258 AEW541 Topotecan Erlotinib PD0332991 Irinotecan PHA665752 17AAG Panobinostat PD0325901 RAF265 ZD6474 LBW242	Nutlin3 TAE684 Nilotinib AZD6244 PF2341066
ANLN	Anillin Actin binding protein	Glomerulosclerosis 8 Focal glomerulosclerosis Liver carcinoma Polycystic Ovary Syndrome Hyalinosis, Segmental Glomerular Sclerocystic Ovaries RDW—Red blood cell distribution width result Red cell distribution width determination Finding of Mean Corpuscular Hemoglobin Systolic Pressure	hsa‐miR‐6807‐3p hsa‐miR‐217‐5p hsa‐miR‐122b‐5p hsa‐miR‐3163 hsa‐miR‐3119 hsa‐miR‐646 hsa‐miR‐5688 hsa‐miR‐495‐3p hsa‐miR‐4255 hsa‐miR‐520 h	TKI258 Paclitaxel AZD0530 L685458 AEW541 Panobinostat Lapatinib PD0325901 PD0332991 Nutlin3 Topotecan PLX4720 RAF265 TAE684 PF2341066 Irinotecan Nilotinib ZD6474	17AAG Erlotinib AZD6244 PHA665752 LBW242 Sorafenib

*Note:* Drugs with resistance are those drugs that could be ineffective to cancer treatment with the upregulation of the corresponding gene target; drugs with sensitivity are drugs potentially showing effectivity to cancer treatment with the upregulation of the corresponding gene target.

### 3.6 Pharmacological Prospects and Therapeutic Targets

The miRDB tool revealed the top 10 miRNAs with the highest target scores for the hub genes associated with HCC (Table 2). These findings suggest that these miRNAs are likely to exert a prominent regulatory influence on the hub genes within the context of HCC. By the use of cancerDP tool we can identify potential drugs against a specific disease taking into account gene expression patterns. In our study, we found that all the HCC related hub genes but EZH2 can be targeted by several drugs, which suggests that they could be interesting candidates for HCC treatment (Table 2).

## 4 Discussion

HCC has an escalating mortality and morbidity rates in the recent years. The development of this cancer can be influenced by a multitude of factors and therefore, understanding the molecular pathways and genes involved in HCC can offer valuable insights for designing effective treatment strategies. Utilizing bioinformatic data analysis tools, we have identified genes that exhibit altered expression in HCC and consequently could influence the dynamics of the associated pathways. Notably, the p53 signaling, pyrimidine metabolism, glutamine metabolism, lysine degradation, ECM‐receptor interaction and cell cycle pathways demonstrate the most pronounced changes in response to altered gene expression. Among the identified hub genes, *RRM2*, *CCNB1*, *EZH2*, and *HMMR* exerts the most substantial effect on these cellular pathways.

Ribonucleotide reductase regulatory subunit M2 (*RRM2*), a gene exhibiting increased expression in liver cancer, holds significant relevance in the field. Extensive research has explored the importance of *RRM2* and its impact on liver cancer, as well as other cancer types by the regulation and modification of proteins, making it a vital component for tumor progression and a potential biomarker for certain cancer [13]. Its involvement extends widely to tumor growth, metastasis, and drug resistance across various cancer types [14, 15]. Numerous clinical studies have been dedicated to *RRM2*, particularly in the context of HCC, revealing that RRM2 is inhibited by the anticancer drug *Sorafenib* in HCC cells [16, 17].

*CCNB1* (Cyclin B1) has been linked to several diseases, including Retinoblastoma and Laryngeal Squamous Cell Carcinoma [18, 19]. Numerous studies have investigated the impact of *CCNB1* on liver cancer, and the present research confirms its potential role in this malignancy. In the liver, *CCNB1* is implicated in the regulation of DNA replication and plays a pivotal role in the cell cycle of HCC cells. Consequently, it holds potential as a diagnostic marker for early‐stage HCC and a target for tailored therapeutic interventions [20]. Furthermore, Chai N et al. described the implications of elevated *CCNB1* expression levels and their effects on the P53 signaling pathway in different cancer types, including pancreatic cancer [21]. Hyaluronan‐mediated motility receptor (*HMMR*) primarily functions in various cellular pathways, including the cell cycle, PLK1 pathway, E2F pathway, ATR pathway, AURORA B pathway, DNA replication, and repair [22]. This protein represents an important biomarker influencing tumor progression and is associated with immune cell infiltration in HCC [23]. Closing the list, but not least important, Enhancer of zeste homolog 2 (*EZH2*), a member of the polycomb group (PcG) genes, acts as an epigenetic regulator that represses gene transcription [24]. Studies have indicated that *EZH2* plays a crucial role in carcinogenesis by suppressing genes, including miRNAs [25]. Interestingly, the potential of *RRM2* and *CCNB1* as biomarkers and therapeutic targets for HBV‐related HCC has been already addressed in depth. Notably, when p53 was knocked down or knocked out in liver cancer cell lines, both p53 and the expression of these two genes decreased [26]. Altogether, *RRM2*, *CCNB1*, *HMMR*, and *EZH2* seemed to be key genes in cancer development. The cancerDP database proves to be an invaluable resource for healthcare professionals, allowing them to make more informed treatment decisions and highlighting potential drug resistance from the disregulation of specific genes. Furthermore, it allows them to consider alternative therapies to counteract cancer resistance or to tailor treatment plans to suit individual patient needs. For example, based on our results, *RRM2* upregulation is related to a reduction of OS in HCC. *Sorafenib* however is provided by cancerDP as drug that can show resistance by upregulation of *RRM2* (Table 2). This apparent contradiction suggests that HCC patients with high levels of *RRM2* could not be best suited for treatment with *Sorafenib*, while patients with low levels of *RRM2* could succeed with the same drug [16, 17].

Finally, in the current work we aimed to investigate the miRNAs that target the HCC hub genes. Although to our best knowledge none of the proposed miRNAs in Table 2 have been investigated in vivo or in vitro in the context of cancer, various studies have attempted to suppress the expression of the HCC hub genes as miRNAs would do. Phase II trials with the drug *Tazemetostat* have demonstrated favorable results in lymphoma patients carrying EZH2 mutations, achieving an impressive 69% objective response rate, while those with wild‐type EZH2 just saw 35% of response rate [27]. In another study, Fang et al. showed that hsa‐microRNA‐411–5p controls proliferation, migration, and invasion in ovarian cancer by targeting the *HMMR* [28], while Junsheng et al. indicated that miRNA‐144 effectively restricts cell proliferation, migration, and invasion in HCC by specifically targeting *CCNB1* [29]. Regarding *RRM2*, miRNA‐582‐3p has been found to act on the regulatory subunit M2 of ribonucleotide reductase, contributing to the inhibition of HCC development by regulating the Wnt/β‐catenin signaling pathway [30]. Intriguingly, one of the subjects that scientists extensively study in the clinical field is “polypharmacology” or “multi‐target therapy,” where a single drug can target multiple factors [31]. In our research, we observed that some miRNAs have the capability to target several hub genes at the same time, such as *ASPM* and *KIF14*, targeted by hsa‐miR‐3646, or hsa‐miR‐548, which could target *ASPM*, *EZH2*, *CCNB1*, and *HMMR* simultaneously. Altogether, these examples illustrate the feasibility of targeting these genes by miRNAs, potentially yielding favorable outcomes. Further research focusing on our identified miRNAs and their therapeutic applications is needed since they could be suitable candidates for HCC treatment.

## 5 Conclusion

Our research has successfully identified a crucial set of genes that play a significant role in the development and progression of liver cancer. These genes, including *RRM2*, *CCNB1*, *EZH2*, and *HMMR*, are intricately involved in regulating essential cellular pathways and signaling cascades that are critical in the pathogenesis of liver cancer. Targeting these genes or the pathways they control is promising for developing innovative therapeutic strategies. However, it is important to note that our findings from bioinformatic analysis need further validation through experimental studies. By bridging the gap between bioinformatic analysis and experimental validation, we can gain deeper insights into the molecular mechanisms underlying liver cancer and pave the way for the development of targeted therapies associated with these hub genes.

## Author Contributions

**Ata Moghimi:** writing – original draft, funding acquisition, investigation, conceptualization, methodology, software, data curation. **Nasrin Bani Hosseinian:** software, methodology, conceptualization, investigation, funding acquisition, writing – original draft, data curation. **Mahdi Mahdipour:** software, formal analysis, methodology, conceptualization, investigation, funding acquisition, writing – original draft. **Ehsan Ahmadpour:** supervision, resources, project administration, writing – review and editing, visualization, validation. **Alberto Miranda‐Bedate:** formal analysis, software, methodology, validation, visualization, writing – review and editing, supervision. **Saeid Ghorbian:** supervision, resources, project administration, formal analysis, software, validation.

## Ethics Statement

This study was supported by a grant from Tabriz University of Medical Sciences under ethical code of IR.TBZMED.VCR.REC.1401.392.

## Conflicts of Interest

The authors declare no conflicts of interest.

## Data Availability

All data are included in the present study.
